# Disturbance-Estimated Adaptive Backstepping Sliding Mode Control of a Pneumatic Muscles-Driven Ankle Rehabilitation Robot

**DOI:** 10.3390/s18010066

**Published:** 2017-12-28

**Authors:** Qingsong Ai, Chengxiang Zhu, Jie Zuo, Wei Meng, Quan Liu, Sheng Q. Xie, Ming Yang

**Affiliations:** 1 School of Information Engineering, Wuhan University of Technology, Wuhan 430070, China; qingsongai@whut.edu.cn (Q.A.); zchengx0508@whut.edu.cn (C.Z.); zuojie@whut.edu.cn (J.Z.); quanliu@whut.edu.cn (Q.L.); s.q.xie@leeds.ac.uk (S.X.); 2 Key Laboratory of Fiber Optic Sensing Technology and Information Processing, Ministry of Education, Wuhan University of Technology, Wuhan 430070, China; 3 School of Electronic and Electrical Engineering, University of Leeds, Leeds LS2 9JT, UK; 4 Faculty of Engineering, Environment and Computing, Coventry University, Coventry CV1 5FB, UK; ab2032@coventry.ac.uk

**Keywords:** parallel robot, ankle rehabilitation, pneumatic muscles, disturbance estimation, adaptive sliding mode control

## Abstract

A rehabilitation robot plays an important role in relieving the therapists’ burden and helping patients with ankle injuries to perform more accurate and effective rehabilitation training. However, a majority of current ankle rehabilitation robots are rigid and have drawbacks in terms of complex structure, poor flexibility and lack of safety. Taking advantages of pneumatic muscles’ good flexibility and light weight, we developed a novel two degrees of freedom (2-DOF) parallel compliant ankle rehabilitation robot actuated by pneumatic muscles (PMs). To solve the PM’s nonlinear characteristics during operation and to tackle the human-robot uncertainties in rehabilitation, an adaptive backstepping sliding mode control (ABS-SMC) method is proposed in this paper. The human-robot external disturbance can be estimated by an observer, who is then used to adjust the robot output to accommodate external changes. The system stability is guaranteed by the Lyapunov stability theorem. Experimental results on the compliant ankle rehabilitation robot show that the proposed ABS-SMC is able to estimate the external disturbance online and adjust the control output in real time during operation, resulting in a higher trajectory tracking accuracy and better response performance especially in dynamic conditions.

## 1. Introduction

The ankle joint plays a key role in maintaining balance during walking [1,2,3]. Recently, there have been an increasing number of people suffering from ankle injuries caused by diseases and accidents. In the US, more than 23,000 cases of ankle sprain injuries happen every day [4]. The postoperative recovery from ankle injury is slow and ineffective while the application of rehabilitation robots is supposed to be possible to solve this problem. Rehabilitation robots can help patients accomplish repetitive training tasks more accurately and effectively without physical therapists’ excessive participation [5,6,7]. Increasing attention has been paid to the robotic rehabilitation that is appropriate to perform repetitive exercises for the recovery from neuromuscular injuries [8].

In the perspective of ankle rehabilitation, parallel robots can produce greater torque as well as achieve multiple movement degrees of freedom (DOFs) [9]. A series of parallel platform-based ankle rehabilitation robots have been developed [10]. Liu et al. [11], Alireza et al. [12], and Mozafar et al. [13] all proposed a 6-DOF ankle rehabilitation robot based on the Stewart platform. However, these robots utilized rigid actuators, such as electric motors or cylinders [14] that cannot achieve soft and compliant interaction with the patients. To overcome the limitations, some researchers started to use pneumatic muscles (PMs) as actuators to drive the ankle rehabilitation robot. PMs have inner compliance, high power/weight ratio [15] and can drive the robot in a safer way, so they have become increasingly popular in the rehabilitation robots [16]. Xie et al. [17,18] designed a four PMs-driven 3-DOF ankle rehabilitation robot with large workspace and good flexibility. Park et al. [19] in Harvard University designed a PMs-driven ankle rehabilitation robot by simulating the human muscle-tendon-ligament model, in which the PMs directly drove the foot to complete dorsiflexion/plantarflexion and inversion/eversion movements. Sawicki et al. [20] also used multiple PMs to provide dorsiflexion and plantar flexion torque for the ankle movement. Patrick et al. [21] designed a 2-DOFs ankle rehabilitation robot driven by three PMs to help patients achieve plantarflexion/dorsiflexion and inversion/eversion movements. 

PMs have strong non-linearity and time-varying properties [22], which may cause difficulties in implementing precise control [23]. In order to solve these problems, a variety of control approaches have been developed. Zhao et al. [24] used neural network to adjust the parameters of PID controller. However, the method has the problems of long response time, poor tracking on desired trajectory and low tracking accuracy in the step response experiment. Zhang et al. [25] proposed a hybrid fuzzy controller to control the elbow exoskeleton robot actuated by PMs. However, this method cannot estimate the external disturbance when chattering happens, resulting in a large overshoot of step response. For the safety of human-robot interaction, Choi et al. [26] proposed a new approach to control the compliance and associated position independently. However, when an external disturbance occurs suddenly, the control method cannot detect the external disturbance quickly and it takes a long time to re-track the desired trajectory. Meng et al. [9] proposed an iterative feedback tuning control method for the repetitive training. However, the actual trajectory changed in a ladder shape because the external disturbance cannot be estimated. Jiang et al. also [27] proposed an adaptive fuzzy control algorithm based on neural network optimization to control the humanoid lower limb device driven by pneumatic muscles. However, this method cannot achieve high-accuracy tracking control and the error would significantly increase when the external load changes. 

During the operation of rehabilitation robot, external disturbances are usually inevitable [28]. To obtain good control performance, the applied disturbance needs to be known exactly. However, external disturbances are often difficult to get accurately [29]. Therefore, one of the reasons why the above control method cannot achieve better control accuracy is that the external disturbance cannot be estimated. It has been recently accepted that the disturbance observer is a good choice to solve this problem [30]. Yang et al. [31] designed an error-feedback controller based on extended state observer to estimate the external disturbances and improve the trajectory tracking accuracy of a PMs-driven robot. Zhu et al. [32] presented an adaptive robust controller based on a pressure observer to control a three PMs-driven robot without pressure sensors. Wu et al. [33] proposed a novel nonlinear disturbance observer-based dynamic surface control (NDOBDSC) and can solve the friction and unknown external disturbances existing in the PM-driven device. Youssif et al. [34] designed a nonlinear disturbance observer (NDO) to estimate the lumped disturbance. Zhang et al. [35] proposed an active disturbance rejection controller for a PM actuator to achieve angle tracking precisely under varying load conditions. Plenty of studies have implied that external disturbance observer can reduce the error and improve the control accuracy effectively. 

On the other hand, since the parallel robot actuated by PMs is a complex high-order nonlinear system, it would be increasingly difficult to develop an accurate control scheme for the system [36]. The backstepping sliding mode control (BS-SMC) can decompose a high-order nonlinear system into several lower order subsystems and design an intermediate virtual controller for each subsystem, which can improve the control performance [37]. In recent years, BS-SMC has attracted the interest of many researchers. Petit et al. [38] used backstepping sliding mode method to control a robot with variable stiffness and achieved satisfactory tracking performance. However, the tracking error would obviously increase if external disturbance occurred. Taheri et al. [39] designed a backstepping sliding mode controller for pneumatic cylinders suitable for wearable robots. The force and stiffness tracking performance were better than the previous pneumatic force-stiffness sliding mode controllers. However, the overshoot of this control scheme was still large and there was no experiment with variable loads. Esmaeili et al. [40] used a backstepping sliding mode controller to achieve balancing and trajectory tracking of Two Wheeled Balancing Mobile Robots (TWBMRs). 

As concluded from the previous studies, there will be excessive overshoots or significantly increased errors when the external disturbance happens. The main reason is that the above methods cannot estimate the external disturbance, and as a result the control output cannot be adjusted in real time. This paper will propose an adaptive backstepping sliding mode control (ABS-SMC) with the capacity to estimate the external disturbance during operation, thus improving the robustness and accuracy of the control method. The ABS-SMC method is applied to a new 2-DOF parallel ankle rehabilitation robot which has been recently developed by us using pneumatic muscles. The controller can also deal with the nonlinearities and uncertainties of the robot system. The rest of this paper is arranged as follows: Section 2 presents mechanism design of the ankle robot. The control strategy is described in Section 3. In Section 4, experiments are carried out to verify the performance of the controller. Section 5 draws conclusion of the paper.

## 2. The Ankle Rehabilitation Robot 

**Figure 1:**
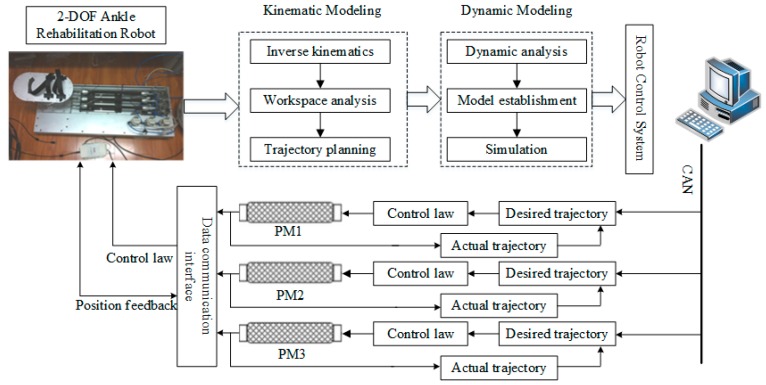
System structure of the ankle rehabilitation robot.

**Figure 2:**
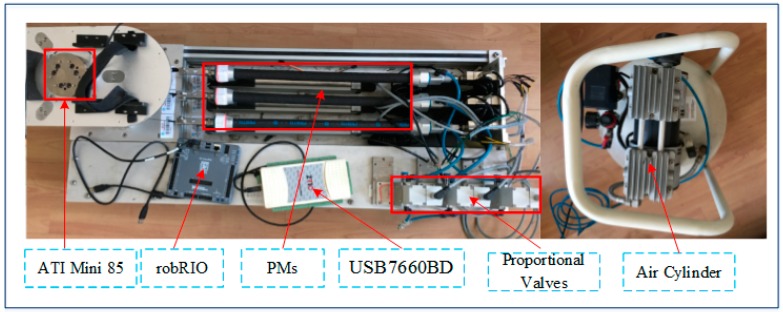
The developed ankle rehabilitation robot driven by PMs.

The complete system of the 2-DOF ankle rehabilitation robot and its hardware configuration are shown in Figure 1 and Figure 2, respectively. The robot consists of a fixed platform, a moving platform, and three pneumatic muscle actuators. The moving platform is equipped with two angle sensors (GONIOMETER SG110) to measure its real-time orientation angle around the X and Y axis. Each pneumatic muscle (FESTO MAS-20-400N) is controlled by an air pressure proportional valve (ITV 2050-212N). The position information of each pneumatic muscle is collected by displacement transducers (MLO-POT-225-TLF). A force/toque sensor (ATI Mini85) is mounted between the platform and the footplate to measure the applied ankle torque. Through the data acquisition card, the sensing data are gathered by robRIO and then transmitted to the host computer. After the D/A conversion of the data, the control signals are input to the corresponding proportional valves to control pneumatic muscles, thus driving the upper platform to move. The ABS-SMC is implemented in the host computer and closed-loop control is realized on LabVIEW.

**Figure 3:**
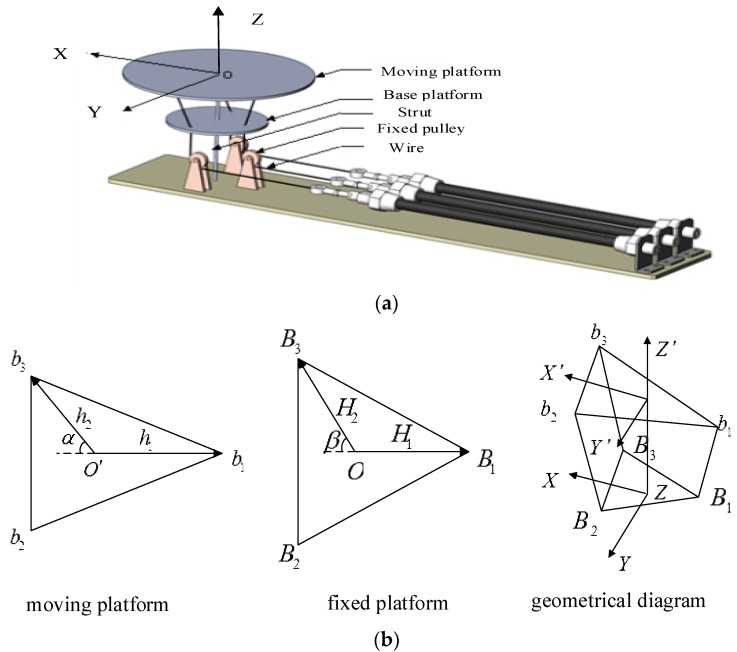
Kinematics of the designed 2-DOF ankle rehabilitation robot: (**a**) structure model, (**b**) geometrical diagram.

Figure 3a,b show the simplified structure and geometrical model of the designed ankle rehabilitation robot. Since the PM can only provide pulling force, the robot must have a redundant actuation mechanism [41]. So the 2-DOF ankle rehabilitation robot is actuated by three pneumatic muscles. The lower fixed platform has three fixed holes, and the wires pass through the holes on the fixed platform. A strut is fixed between the fixed platform and the moving platform (end-effector). The Hooke joints between these two platforms guarantee that the robot can only move at two orientations. When the muscles’ lengths change, the platform can be controlled to work on two orientations. In order to reduce the height of the robot and make it easier for human usage, three PMs are placed in the horizontal direction, using three fixed pulleys to change the direction of actuating forces. In this case, the overall height of the robot is only 0.3 m.

In order to control the robot end-effector to track a predefined trajectory for ankle movement training, the robot kinematic model must be studied [42], using which the joint space displacements can be determined from the end-effector orientation. As shown in Figure 3b, $b_{1}b_{2}b_{3}$ and $B_{1}B_{2}B_{3}$ represent the moving platform and the fixed platform, respectively. The vectors that connect the moving platform and the fixed platform can be written as $b_{1}B_{1}$, $b_{2}B_{2}$ and $b_{3}B_{3}$. $O-X^{\prime }Y^{\prime }Z^{\prime }$ and O−XYZ are coordinate system of the moving platform and the fixed platform, respectively. A space vector in the moving coordinate can be transformed to the fixed via rotation matrix, which is widely used to establish inverse kinematics of the parallel rehabilitation robot [43]. Here $\alpha \;=\;50^{o}$, $\beta \;=\;80^{o}$, $h_{1}=0.07\;m$, $h_{2}=0.08\;m$, $H_{1}=0.05\;m$, $H_{2}=0.06\;m$. The rotation matrix can be expressed as:

(1) $$T=T(y,\phi )T(x,\theta )=\left[\begin{array}{ccc} \cos\phi & \sin\phi \sin\theta & \sin\phi \cos\theta \\ 0 & \cos\theta & -\sin\theta \\ -\sin\phi & \cos\phi \sin\theta & \cos\phi \cos\theta \end{array}\right].$$

The solution of $b_{1}B_{1}$, $b_{2}B_{2}$ and $b_{3}B_{3}$ is necessary for robot control and workspace analysis. It can be obtained by using the inverse kinematics. The link’s length of this parallel robot is:

(2) $$l_{i}={\left|L\right|}_{i}=\left|Tr_{b_{i}}^{'}+P-r_{B_{i}}\right|i=1,2,3$$

where $L_{i}$ is the vector from $B_{i}$ to $b_{i}$, P is the vector from O to $O^{\prime }$, $r_{b_{i}}^{\prime }$ is the vector from $O^{\prime }$ to $b_{i}\left(i=1,2,3\right)$ and $r_{B_{i}}^{\prime }$ is the vector from O to $B_{i}\left(i=1,2,3\right)$.

The dynamic model of the robot describes the relationship between the output torque and the desired angle as well as angular velocity [44]. The dynamics model is also the foundation of sliding mode control [45]. Define $q={\left[\begin{array}{ccc} \theta & \varphi & \phi \end{array}\right]}^{T}={\left[\begin{array}{ccc} \theta & \varphi & 0 \end{array}\right]}^{T}$ as the generalized coordinates of the robot’s moving platform, thus the generalized speed of the moving platform is shown in Equation (3).

(3) $$\omega =\tilde{E}\cdot \left[\begin{array}{c} \dot{\theta }\\ \dot{\varphi }\\ 0 \end{array}\right]=\left[\begin{array}{ccc} \cos\varphi & 0 & 0\\ 0 & 1 & 0\\ -\sin\varphi & 0 & 1 \end{array}\right]\left[\begin{array}{c} \dot{\theta }\\ \dot{\varphi }\\ 0 \end{array}\right].$$

Lagrange’s equation is suitable for the complete system and it can solve the complex system dynamic equation in a simpler way [46]. So we use the Lagrange’s equation to establish the dynamic equation of the moving platform:

(4) $$M(q)\ddot{q}+C(q,\dot{q})\dot{q}+G(q)=\tau +\tau_{d},$$

where M(q), $C(q,\dot{q})$ and G(q) represent the robot inertia matrix, the Coriolis centrifugal force matrix and the gravity matrix, τ is the robot torque and $\tau_{d}$ is the external disturbance torque. $\tau_{d}$ is mainly composed of human applied torque and the friction. The parameters in Equation (4):

(5) $$\begin{array}{l} M(q)=TI_{P}T^{T}\\ C(q,\dot{q})\dot{q}=\tilde{\omega }TTI_{P}T^{T}\\ G(q)=-m{\tilde{T}}_{r_{m}}g \end{array},$$

where m is the mass of the moving platform, $I_{p}$ is the rotational inertia of the moving platform, $r_{m}$ is the position vector of the moving platform centroid, $T_{r_{m}}=Tr_{m}$ and ${\tilde{T}}_{r_{m}}$ is the spiral matrix of $T_{r_{m}}$. According to the formula, the driving force of each pneumatic muscle can be obtained, and finally to realize the accurate trajectory tracking of the robot platform.

## 3. Control Strategy

### 3.1. Backstepping Sliding Mode Control

The basic idea of backstepping design method is to decompose the complex nonlinear system into subsystems with lower orders, and then design Lyapunov function and intermediate virtual control for each subsystem [47]. Based on Equation (4), the controlled object model can be defined as

(6) $$\{\begin{array}{c} {\dot{q}}_{1}=q_{2}\\ {\dot{q}}_{2}=-M^{-1}Cq_{2}+M^{-1}\tau -M^{-1}G+M^{-1}\tau_{d} \end{array},$$

where $q_{1}=q$, q is the actual trajectory.

Assuming the desired position $q_{d}$, the controller can be designed by the following two steps:

***Step 1*:** Define the tracking error $e_{1}=q_{1}-q_{d}$, then ${\dot{e}}_{1}={\dot{q}}_{1}-{\dot{q}}_{d}=q_{2}-{\dot{q}}_{d}$, and define the Lyapunov function as

(7) $$V_{1}=\frac{1}{2}e_{1}^{T}e_{1}.$$

So

(8) $${\dot{V}}_{1}=e_{1}^{T}{\dot{e}}_{1}=e_{1}^{T}\left(q_{2}-{\dot{q}}_{d}\right).$$

Define

(9) $$q_{2}=e_{2}+{\dot{q}}_{d}-c_{1}e_{1},$$

where $c_{1}>0$, $e_{2}$ is a virtual control law. From Equation (9), we can obtain

(10) $$\begin{array}{ll} {\dot{e}}_{1} & ={\dot{q}}_{1}-{\dot{q}}_{d}\\ & =q_{2}-{\dot{q}}_{d}+c_{1}e-c_{1}e_{1}\\ & =e_{2}-c_{1}e_{1} \end{array}.$$

From Equations (8) and (10) we can obtain

(11) $${\dot{V}}_{1}=e_{1}^{T}{\dot{e}}_{1}=e_{1}^{T}e_{2}-c_{1}e_{1}^{T}e_{1}.$$

If $e_{2}=0$, ${\dot{V}}_{1}=-c_{1}e_{1}^{T}e_{1}=-c_{1}{\left({\left\|e_{1}\right\|}_{2}\right)}^{2}\leq 0$. So it is necessary to further design the control law.

***Step 2*:** Define the switch function as

(12) $$s=k_{1}e_{1}+e_{2},$$

where $k_{1}>0$. Taking Equation (10) into (12), we can obtain

(13) $$s=k_{1}e_{1}+{\dot{e}}_{1}+c_{1}e_{1}=(k_{1}+c_{1})e_{1}+{\dot{e}}_{1}.$$

The Lyapunov function is

(14) $$V_{2}=\frac{1}{2}e_{1}^{T}e_{1}+\frac{1}{2}s^{T}s.$$

From Equation (14) we can obtain

(15) $$\begin{array}{ll} {\dot{V}}_{2}= & e_{1}^{T}{\dot{e}}_{1}+s^{T}\dot{s}\\ & =e_{1}^{T}e_{2}-c_{1}e_{1}^{T}e_{1}+s^{T}(k_{1}(e_{2}-c_{1}e_{1})-M^{-1}C(e_{2}+{\dot{q}}_{d}-c_{1}e_{1})\\ & +M^{-1}\tau +M^{-1}\tau_{d}-M^{-1}G-{\ddot{q}}_{d}+c_{1}{\dot{e}}_{1}) \end{array}.$$

So the control law can be written as

(16) $$\tau_{BS-SMC}=\tau_{eq}+M\Delta \tau ,$$

where

(17) $$\begin{array}{c} \tau_{eq}=M(-k_{1}(e_{2}-c_{1}e_{1})+M^{-1}C(e_{2}+{\dot{q}}_{d}-c_{1}e_{1})+M^{-1}G+{\ddot{q}}_{d}-c_{1}{\dot{e}}_{1})\\ \Delta \tau =-h(s+\beta \mathrm{sgn}(s)) \end{array}.$$

where h and β are the parameters of exponential reaching law. They can determine the speed and time of the moving point approaching to the sliding surface.

### 3.2. Adaptive Backstepping Sliding Mode Control

The proposed ABS-SMC can estimate the external disturbance by establishing an disturbance observer [48]. Assuming that the external disturbance observer is ${\hat{\tau }}_{d}$.

Define

(18) $$Q=\left[\begin{array}{c} q_{1}\\ q_{2} \end{array}\right].$$

So

(19) $$\begin{array}{ll} \dot{Q} & =\left[\begin{array}{c} {\dot{q}}_{1}\\ {\dot{q}}_{2} \end{array}\right]=\left[\begin{array}{c} q_{2}\\ {\dot{q}}_{2} \end{array}\right]\\ & =\left[\begin{array}{c} q_{2}\\ -M^{-1}Cq_{2}-M^{-1}G+M^{-1}\tau +M^{-1}\tau_{d} \end{array}\right] \end{array},$$

Equation (19) can be rewritten as:

(20) $$\begin{array}{ll} \dot{Q} & =\left[\begin{array}{c} q_{2}\\ -M^{-1}Cq_{2}-M^{-1}G \end{array}\right]+\left[\begin{array}{c} 0\\ M^{-1} \end{array}\right]\tau +\left[\begin{array}{c} 0\\ M^{-1} \end{array}\right]\tau_{d}\\ & =f_{1}(Q)+f_{2}(Q)\tau +f_{2}(Q)\tau_{d} \end{array},$$

where

(21) $$f_{1}(Q)=\left[\begin{array}{c} q_{2}\\ -M^{-1}Cq_{2}-M^{-1}G \end{array}\right];f_{2}(Q)=\left[\begin{array}{c} 0\\ M^{-1} \end{array}\right],$$

The disturbance observer is designed based on the difference between estimated output and actual output. Equation (20) can be rewritten as

(22) $$f_{2}(Q)\tau_{d}=\dot{Q}-f_{1}(Q)-f_{2}(Q)\tau ,$$

So the disturbance observer is designed:

(23) $${\dot{\hat{\tau }}}_{d}=\Gamma (\dot{Q}-f_{1}(Q)-f_{2}(Q)\tau -f_{2}(Q){\hat{\tau }}_{d}),$$

Define vector $z={\hat{\tau }}_{d}-p(Q)$. The observer gain can be expressed as Γ=∂p(Q)/∂Q. Let

(24) $$\Gamma =\left[\begin{array}{cc} \xi_{2} & \xi_{2} \end{array}\right],\xi_{1}>0,\xi_{2}>0$$

(25) $$p(Q)=\xi_{1}q_{1}+\xi_{2}q_{2}=\xi_{1}q+\xi_{2}\dot{q}.$$

(26) $$\dot{z}={\dot{\hat{\tau }}}_{d}-\dot{p}(Q).$$

Substituting Equations (23) and (25) into (26),

(27) $$\begin{array}{ll} \dot{z} & ={\dot{\hat{\tau }}}_{d}-\dot{p}(Q)\\ & =\Gamma (-f_{1}(Q)-f_{2}(Q)\tau -f_{2}(Q)(z+p(Q)))\\ & +\left[\begin{array}{cc} \xi_{1} & \xi_{2} \end{array}\right]{\left[\begin{array}{cc} \dot{q} & \ddot{q} \end{array}\right]}^{T}-\xi_{1}\dot{q}-\xi_{2}\ddot{q}\\ & =\Gamma (-f_{1}(Q)-f_{2}(Q)\tau -f_{2}(Q)(z+p(Q))) \end{array}.$$

Let ${\tilde{\tau }}_{d}=\tau_{d}-{\hat{\tau }}_{d}$. When the disturbance varies slowly relative to the observer dynamics, which is commonly assumed in observer design [48,49], it is reasonable that ${\dot{\tau }}_{d}=0$, so we have

(28) $${\dot{\tilde{\tau }}}_{d}+{\dot{\hat{\tau }}}_{d}=0.$$

Substituting Equations (24) and (25) into (28),

(29) $$\begin{array}{l} 0={\dot{\tilde{\tau }}}_{d}+\Gamma (\dot{Q}-f_{1}(Q)-f_{2}(Q)\tau -f_{2}(Q){\hat{\tau }}_{d})\\ ={\dot{\tilde{\tau }}}_{d}+\Gamma (f_{2}(Q)\tau_{d}-f_{2}(Q){\hat{\tau }}_{d})={\dot{\tilde{\tau }}}_{d}+\Gamma f_{2}(Q){\tilde{\tau }}_{d} \end{array}.$$

Substituting Equation (21) into (27), the disturbance observer can be written as

(30) $$\begin{array}{l} {\hat{\tau }}_{d}=z+p(Q)\\ \dot{z}=-(\xi_{1}\dot{q}+\xi_{2}M^{-1}(-C\dot{q}-G+\tau )+\xi_{2}M^{-1}(z+\xi_{1}q+\xi_{2}\dot{q})) \end{array}.$$

Based on Equations (17) and (30), the adaptive control law can be written as

(31) $$\begin{array}{ll} \tau_{ABS-SMC}= & M(-k_{1}(e_{2}-c_{1}e_{1})+M^{-1}C(e_{2}+{\dot{q}}_{d}-c_{1}e_{1})+M^{-1}G\\ & -M^{-1}{\hat{\tau }}_{d}+{\ddot{q}}_{d}-c_{1}{\dot{e}}_{1}-h(s+\beta \mathrm{sgn}(s))) \end{array}.$$

**Figure 4:**
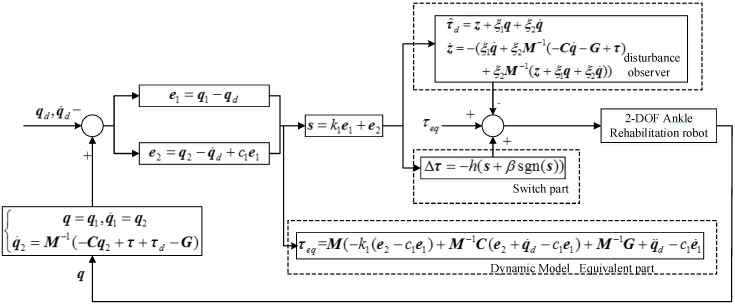
Implementation of ABS-SMC for the ankle rehabilitation robot.

According to these, the proposed ABS-SMC controller for the developed ankle rehabilitation robot with external disturbance in practice can be implemented based on the diagram in Figure 4, in which the controller observer can adaptively estimate the external disturbance.

### 3.3. Stability Analysis

To prove the stability of a closed-loop system, Lyapunov function is commonly used [28,29,50], through which we firstly prove that the estimation error of disturbance is bounded.

**Remark** **1.** *For the dynamic model in (4) and the disturbance observer in (29), the estimation error* ${\tilde{\tau }}_{d}$ *is bounded.*

**Proof.**  Define a Lyapunov function $V_{3}$ as follows:

(32) $$V_{3}=\frac{1}{2}{\tilde{\tau }}_{d}^{T}{\tilde{\tau }}_{d}.$$

Substituting Equations (21) and (24) into (23):

(33) $$\begin{array}{ll} {\dot{\hat{\tau }}}_{d} & =\left[\begin{array}{cc} \xi_{1} & \xi_{2} \end{array}\right]\left(\left[\begin{array}{c} {\dot{q}}_{1}\\ {\dot{q}}_{2} \end{array}\right]-\left[\begin{array}{c} q_{2}\\ -M^{-1}Cq_{2}-M^{-1}G \end{array}\right]-\left[\begin{array}{c} 0\\ M^{-1} \end{array}\right]\tau -\left[\begin{array}{c} 0\\ M^{-1} \end{array}\right]{\hat{\tau }}_{d}\right)\\ & =\xi_{2}(\ddot{q}+M^{-1}Cq_{2}+M^{-1}G-M^{-1}\tau -M^{-1}{\hat{\tau }}_{d}) \end{array}.$$

Substituting Equation (6) into (33):

(34) $${\dot{\hat{\tau }}}_{d}=\xi_{2}(M^{-1}\tau_{d}-M^{-1}{\hat{\tau }}_{d})=\xi_{2}M^{-1}{\tilde{\tau }}_{d}.$$

So

(35) $${\tilde{\tau }}_{d}^{T}{\dot{\tilde{\tau }}}_{d}={\tilde{\tau }}_{d}^{T}({\dot{\tau }}_{d}-{\dot{\hat{\tau }}}_{d})=-{\tilde{\tau }}_{d}^{T}{\dot{\hat{\tau }}}_{d}.$$

Substituting Equation (34) into (35), we have

(36) $${\tilde{\tau }}_{d}^{T}{\dot{\tilde{\tau }}}_{d}=-\xi_{2}{\tilde{\tau }}_{d}^{T}M^{-1}{\tilde{\tau }}_{d}.$$

Because $M^{-1}$ is a positive definite matrix and $\xi_{2}>0$, then

(37) $${\dot{V}}_{3}={\tilde{\tau }}_{d}^{T}{\dot{\tilde{\tau }}}_{d}\leq 0.$$

This indicates that the designed disturbance observer can track external disturbance, which means the estimation error ${\tilde{\tau }}_{d}$ is bounded, so Remark 1 is proved to be correct.

Then, we prove the stability of the combined system. As the robot moves within a confined space, the inertia matrix M is bounded and positive definite so $M^{-1}$ exists and is bounded,

(38) $${\left\|M^{-1}{\tilde{\tau }}_{d}\right\|}_{1}={\left\|M^{-1}\tau_{d}-M^{-1}{\hat{\tau }}_{d}\right\|}_{1}\leq \delta .$$

$\hat{\delta }$ is the estimated value of δ. Then define:

(39) $$\dot{\hat{\delta }}=\gamma {\left\|s\right\|}_{1}.$$

where γ>0 [51,52]. □

**Remark** **2.** *As long as the parameters are appropriately set, the closed-loop system is stable for disturbance observer in (30) and control law in (31)*.

**Proof.**  The Lyapunov function is defined as

(40) $$V=V_{2}+\frac{1}{2\gamma }{\tilde{\delta }}^{2}+\frac{1}{2}{\tilde{\tau }}_{d}^{T}{\tilde{\tau }}_{d}.$$

where $\tilde{\delta }=\delta -\hat{\delta }$.

From Equation (40), we can get

(41) $$\dot{V}={\dot{V}}_{2}+\frac{1}{\gamma }\tilde{\delta }\dot{\tilde{\delta }}+{\tilde{\tau }}_{d}^{T}{\dot{\tilde{\tau }}}_{d}.$$

Substituting Equation (31) into (15), we can get

(42) $$\begin{array}{ll} {\dot{V}}_{2}+\frac{1}{\gamma }\tilde{\delta }\dot{\tilde{\delta }} & =e_{1}^{T}e_{2}-c_{1}e_{1}^{T}e_{1}+s^{T}M^{-1}{\tilde{\tau }}_{d}-hs^{T}s-h\beta {\left\|s\right\|}_{1}-\frac{1}{\gamma }\tilde{\delta }\dot{\hat{\delta }}\\ & \leq e_{1}^{T}e_{2}-c_{1}e_{1}^{T}e_{1}+\delta {\left\|s\right\|}_{1}-hs^{T}s-h\beta {\left\|s\right\|}_{1}-\tilde{\delta }{\left\|s\right\|}_{1}\\ & \;=e_{1}^{T}e_{2}-c_{1}e_{1}^{T}e_{1}-hs^{T}s+(\delta -\tilde{\delta }-h\beta ){\left\|s\right\|}_{1} \end{array}.$$

Let $h\beta =\hat{\delta }=\int \gamma {\left\|s\right\|}_{1}dt$. Equation (42) can be rewritten as:

(43) $$\begin{array}{ll} {\dot{V}}_{2}+\frac{1}{\gamma }\tilde{\delta }\dot{\tilde{\delta }} & \leq e_{1}^{T}e_{2}-c_{1}e_{1}^{T}e_{1}-hs^{T}s+(\delta -\tilde{\delta }-h\beta ){\left\|s\right\|}_{1}\\ & =e_{1}^{T}e_{2}-c_{1}e_{1}^{T}e_{1}-hs^{T}s+(\delta -\tilde{\delta }-\hat{\delta }){\left\|s\right\|}_{1}\\ & =e_{1}^{T}e_{2}-c_{1}e_{1}^{T}e_{1}-hs^{T}s \end{array}.$$

Define $e=\left[\begin{array}{cc} e_{1}^{T} & e_{2}^{T} \end{array}\right]$, $e^{T}=\left[\begin{array}{c} e_{1}\\ e_{2} \end{array}\right]$, and $Β=\left[\begin{array}{cc} c_{1}+hk_{1}^{2} & hk_{1}-\frac{1}{2}\\ hk_{1}-\frac{1}{2} & h \end{array}\right]$.

Then

(44) $$\begin{array}{ll} eΒe^{T} & =\left[\begin{array}{cc} e_{1}^{T} & e_{2}^{T} \end{array}\right]\left[\begin{array}{cc} c_{1}+hk_{1}^{2} & hk_{1}-\frac{1}{2}\\ hk_{1}-\frac{1}{2} & h \end{array}\right]\left[\begin{array}{c} e_{1}\\ e_{2} \end{array}\right]\\ & =c_{1}e_{1}^{T}e_{1}-e_{1}^{T}e_{2}+hk_{1}^{2}e_{1}^{T}e_{1}+hk_{1}e_{1}^{T}e_{2}+hk_{1}e_{2}^{T}e_{1}+he_{2}^{T}e_{2}\\ & =c_{1}e_{1}^{T}e_{1}-e_{1}^{T}e_{2}+hs^{T}s \end{array}.$$

Substituting Equation (44) into (43):

(45) $${\dot{V}}_{2}+\frac{1}{\gamma }\tilde{\delta }\dot{\tilde{\delta }}\leq -eΒe^{T}.$$

If we make Β be a positive definite matrix, then

(46) $${\dot{V}}_{2}+\frac{1}{\gamma }\tilde{\delta }\dot{\tilde{\delta }}\leq -e^{T}Βe\leq 0.$$

Because

(47) $$\begin{array}{ll} \left|B\right| & =h(c_{1}+hk_{1}^{2})-{\left(hk_{1}-\frac{1}{2}\right)}^{2}\\ & =h(c_{1}+k_{1})-\frac{1}{4} \end{array}.$$

By appropriately setting h, $c_{1}$, $k_{1}$, we can make |B|>0, so that B is a positive definite matrix and guarantee ${\dot{V}}_{2}+\frac{1}{\gamma }\tilde{\delta }\dot{\tilde{\delta }}\leq 0$.

From Equation (37), we can get

(48) $${\tilde{\tau }}_{d}^{T}{\dot{\tilde{\tau }}}_{d}\leq 0.$$

Substituting Equations (46) and (48) into (41):

(49) $$\dot{V}={\dot{V}}_{2}+\frac{1}{\gamma }\tilde{\delta }\dot{\tilde{\delta }}+{\tilde{\tau }}_{d}^{T}{\dot{\tilde{\tau }}}_{d}\leq 0.$$

Therefore, as long as the mentioned parameters are appropriately set, we can ensure the system be stable. In this way, Remark 2 is proved to be correct. □

## 4. Experimental and Results Discussion

In order to confirm the performance of the proposed control method, experiments were carried out on the 2-DOF ankle rehabilitation robot. The experiments can be divided into four groups: (1) step response experiment; (2) sine trajectory tracking experiment (without subject); (3) robustness test with human subjects; and (4) sudden external disturbance experiment. BS-SMC has been widely used in recent years and achieved good control performance [38,39,40], so we conduct the experiments to compare the proposed control method with BS-SMC to verify its control capacity and advantages.

### 4.1. Step Response

**Figure 5:**
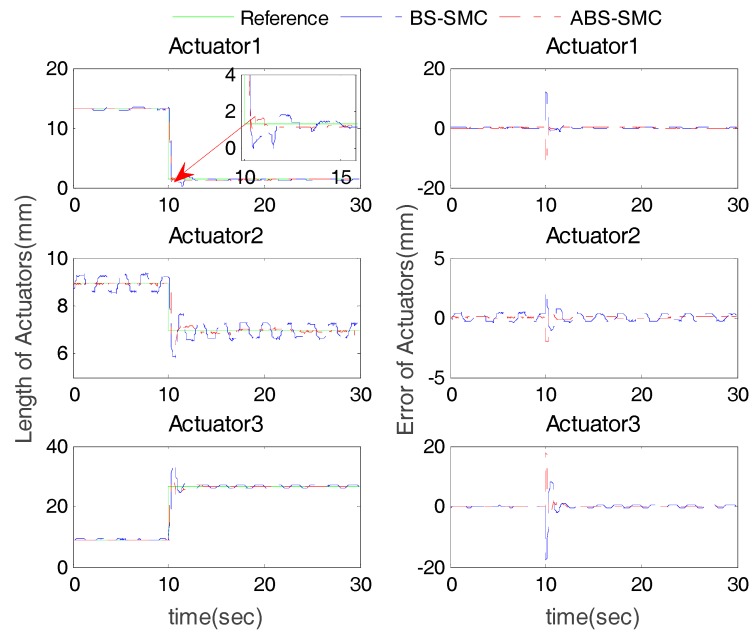
Actuator position tracking results and errors in step response experiment with robot controlled by BS-SMC and ABS-SMC respectively.

To simulate step response, the moving platform was firstly set to its initial pose (*θ* = 0°, *φ* = 0°). Then, at t = 10 s, the expected position of the moving platform was set as *θ* = 10° and *φ* = 10°. The experimental results of both BS-SMC and ABS-SMC are shown in Figure 5.

Figure 5 shows the step response of three PMs under different control methods. It can be seen that both the proposed ABS-SMC and BS-SMC were able to generate delay less than 0.5 s, but the ABS-SMC reached the desired trajectory more quickly after a short shock. The response time of the proposed control method was 1 s while that of the BS-SMC was about 1.5 s. In addition, there was always vibration existing near the desired trajectory in the BS-SMC experiment, while the proposed ABS-SMC could effectively reduce chattering and guaranteed the operation safety. Moreover, the overshoot of ABS-SMC was significantly smaller than that of BS-SMC. For example, the tracking overshoot of Actuator 3 was about 5 mm when controlled by ABS-SMC. If the overshoot is too large, the patient’s foot may have to rotate at a large angle in a short time, which may cause the secondary injury to the patient. On the other hand, after the system reached the steady state, the error of the ABS-SMC was smaller than 0.5 mm while the maximum error of the BS-SMC was 2 mm.

### 4.2. Sine Trajectory Tracking Experiment (without Subject)

**Figure 6:**
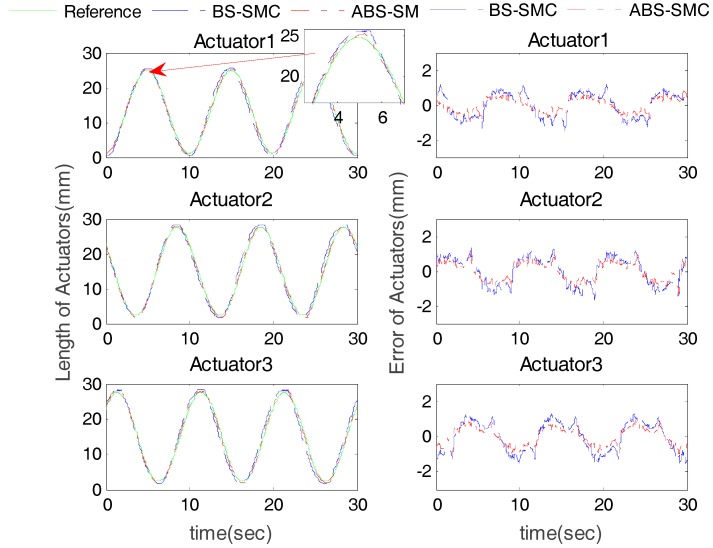
Actuator position tracking results (without subject).

**Figure 7:**
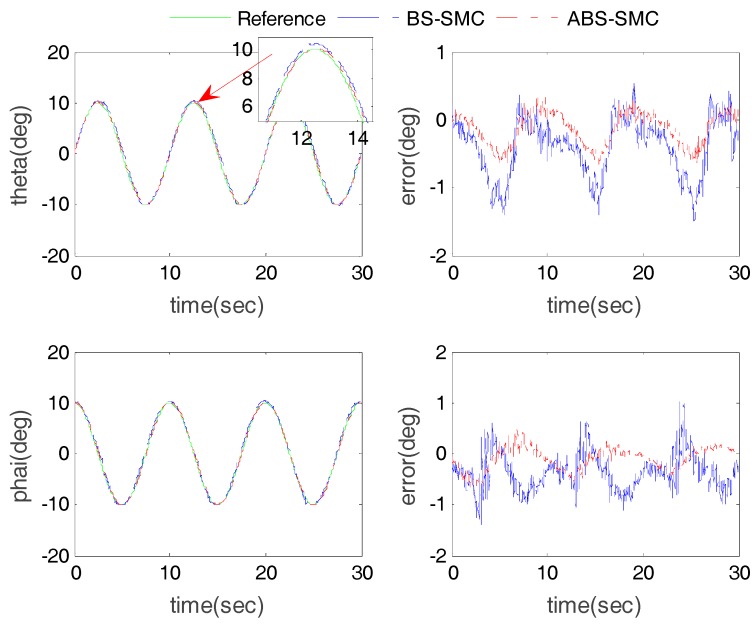
Robot end-effector angle tracking results (without subject).

The desired trajectory was set θ = 10sin(2πft)(deg), φ = 10cos(2πft)(deg), f=10 Hz. The results of sine trajectory tracking with no subject involved (load = 0) are shown in Figure 6 and Figure 7. From Figure 6, we can see that the proposed method had higher control accuracy and smaller chattering than BS-SMC, due to its ability to compensate the external disturbance, which can effectively guarantee the safety and stability of the rehabilitation operations. In order to further quantitatively compare the performance between ABS-SMC and BS-SMC, maximum error (ME) and average error (AE) of the robot control results were calculated for statistical evaluation. Table 1 shows the position tracking errors of the two control methods. Taking Actuator 1 as an example, for the proposed control method, the ME and AE were 0.84 mm and 0.39 mm respectively, while the ME and AE of BS-SMC were 1.48 mm and 0.64 mm. Compared with BS-SMC, the ME and AE of ABS-SMC were reduced by about 43% and 40% respectively. In Table 2, the ME (0.69°) and AE (0.19°) of the rotation angle around X-axis were reduced by 53% and 70%, compared with BS-SMC (1.48° and 0.57°). Compared with BS-SMC, the proposed ABS-SMC cannot only improve the position control accuracy, but also has a lower chattering level attributing to its ability of disturbance estimation. 

### 4.3. Robustness Test with Human Subjects

**Figure 8:**
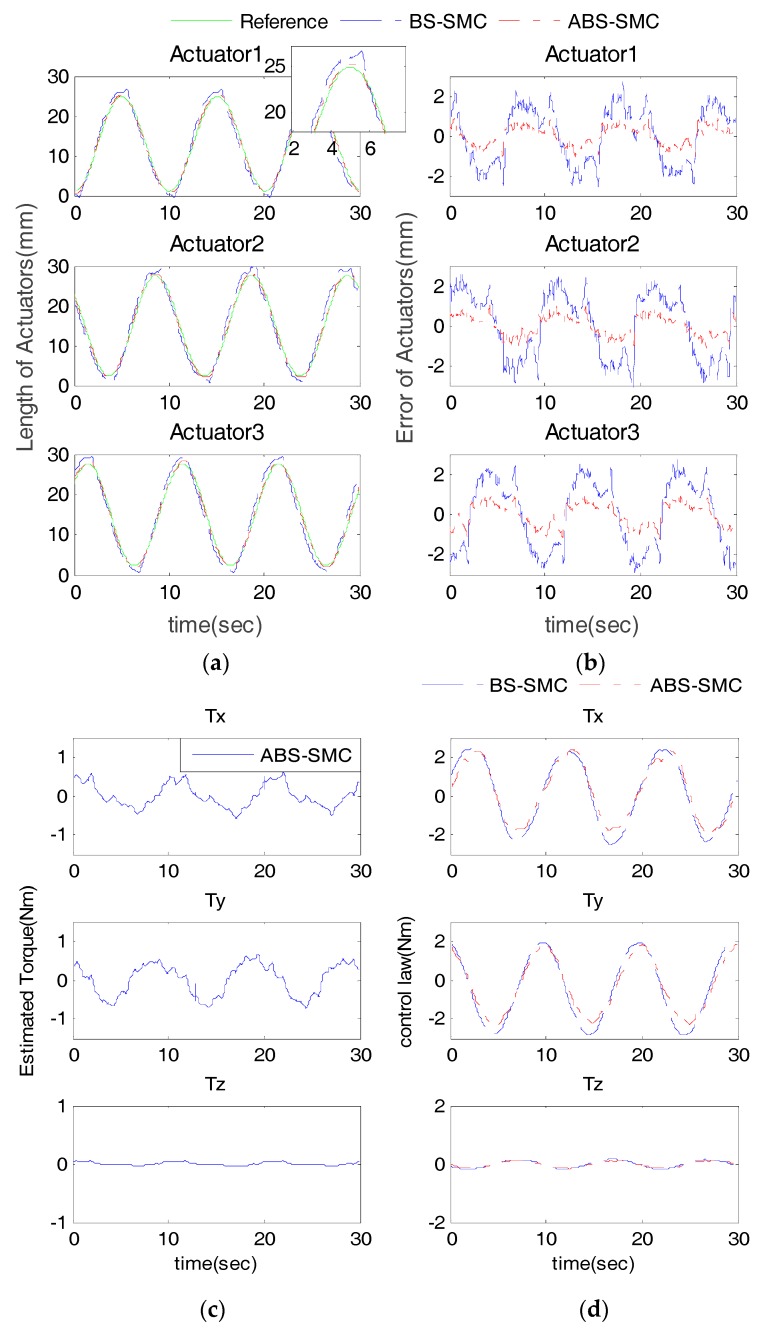
Actuator position tracking results with subject 1: (**a**) actuator position tracking results; (**b**) the actuator tracking errors; (**c**) the estimated external torque (using ABS-SMC) and (**d**) the control output tuning processing via ABS-SMC disturbance estimation.

**Figure 9:**
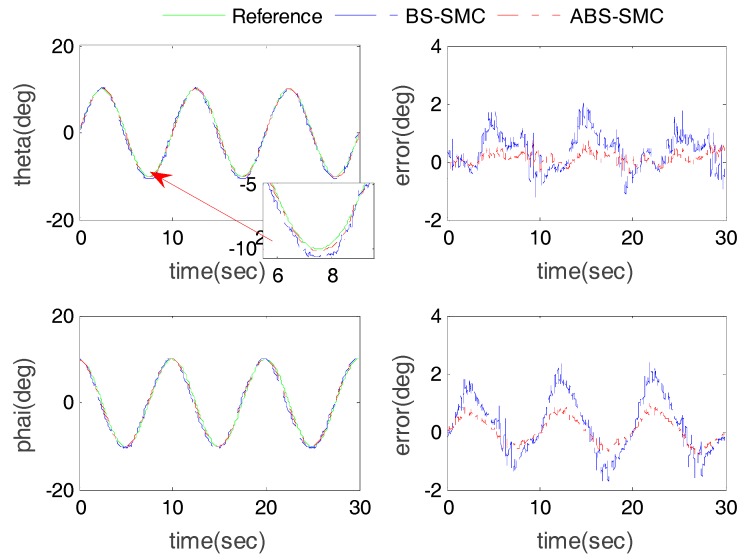
End-effector angle tracking results with subject 1.

In order to verify the robustness of the proposed controller, especially when interacting with human users, five healthy subjects were involved in the experiment. The information of all subjects is shown in Table 3. The participants were instructed to fix their right foot on the robot moving platform so that they can follow the moving platform for passive training. This trial has been approved by the Human Participants Ethics Committees from Wuhan University of Technology, China and written informed consent was obtained from each participant. The experimental results were compared with BS-SMC to verify its superior ability by taking advantage of external disturbance estimation. We take Subject 1 as an example with results shown in Figure 8 and Figure 9.

The results of the sine wave tracking with Subject 1 are shown in Figure 8 and Figure 9. Compared with BS-SMC, we can see that proposed control method has smaller tracking errors. In the case of Actuator 1, as shown in Table 1 and Table 4, when the ABS-SMC was applied to the robot, compared to the experiment without subject, the ME and AE of position tracking result increased by about 0.26 mm and 0.04 mm only. However, when BS-SMC was used, the ME and AE increased by 1.23 mm and 0.66 mm. Comparing Table 2 and Table 5, taking the rotation angle around X axis as an example, in the use of ABS-SMC and when subject participated, the ME and AE only increased by about 0.21° and 0.01°, but the ME and AE increased by 0.56° and 0.10° when using BS-SMC. 

In Figure 8c, the desired trajectory was sinusoidal, so the torque applied by the subject to the moving platform showed a similar pattern. ABS-SMC regarded the exerted force as an external disturbance, thus the estimated external disturbance torque also revealed similar sine changes. On the other hand, it can be seen from Figure 8d that the control law of the proposed ABS-SMC was quite different from that of the BS-SMC, especially when it reached the extreme point. This is because the external disturbance reached the maximum at the extreme point of the control law. It can also be noticed that the estimated external disturbance of Z-axis was much smaller than X and Y axes. This is because the designed robot cannot rotate around the Z-axis. The ideal Z-axis torque should be zero, but in practice the moving platform still has a slight rotation in the Z-axis. 

**Figure 10:**
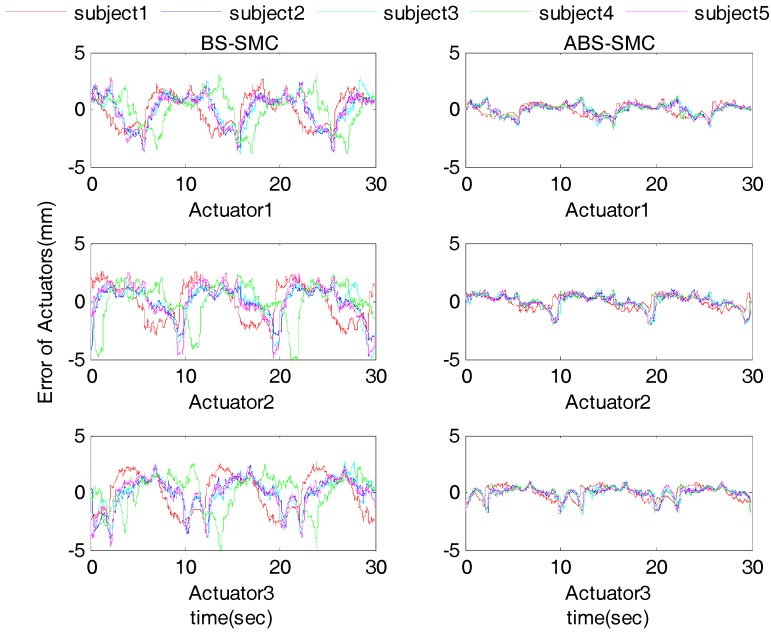
Actuator tracking error results with five subjects.

Figure 10 further shows the errors of three actuators with all five participants. We can see that the proposed ABS-SMC is able to obtain smaller errors which also changed more smoothly. It can be again validated that the proposed ABS-SMC is able to obtain better robustness. The statistical details in Table 4 and Table 5 indicate the robustness of the ABS-SMC scheme for its adaptability to different subjects with varying capabilities. When different subjects involved, the actuators’ ME changed very slightly. The minimum ME was 1.10 mm and the maximum 2.07 mm. The change of AE was also small (0.37~0.49 mm). When using BS-SMC to control the robot, the ME ranged 2.71~5.30 mm, and the AE ranged 1.14~1.56 mm; therefore, the stability and control accuracy of ABS-SMC were better than BS-SMC, which could adapt to different people’s rehabilitation training. Therefore, we can conclude that the ABS-SMC has a better robustness as it estimates the exerted disturbance and adjusts the control law in real time, resulting in higher control accuracy and reduced chattering.

### 4.4. Sudden External Disturbance

**Figure 11:**
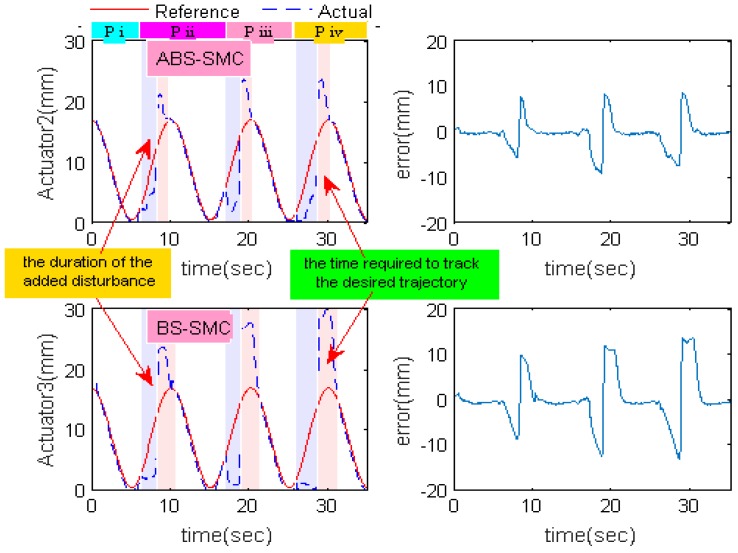
Actuator trajectory tracking results with abrupt disturbances.

To further confirm the anti-interference ability of the proposed ABS-SMC, a certain resistance was applied on the 2-DOFs ankle rehabilitation robot. During different training cycles, the strength and duration of the resistance are shown in Table 6 and the experimental results are compared with BS-SMC. It can be seen that the trajectories of the actuator 2 and 3 were exactly the same when the trajectory of the moving platform is θ = 0°, φ = 10cos(2πft)°. In order to ensure the applied force consistent for the two control methods comparison, the ABS-SMC was used to control the actuator 1 and actuator 2, while the BS-SMC was used to control the actuator 3 of the rehabilitation robot. The experimental results are shown in Figure 11.

Figure 11 shows the trajectory tracking curve after applying sudden disturbances. It can be seen that the time required for ABS-SMC to track the desired trajectory was about 1.53 s and the maximum error was about 7.50 mm in phase ii, while the time required for the BS-SMC was 2.39 s and the maximum error 9.61 mm. In phase iii, compared with phase ii, the time that the proposed ABS-SMC required to tracks the desired trajectory only increased by 3.92% and the maximum error increased by 7.60%. However, the required time and maximum error increased by 7.60% and 20.9% respectively in BS-SMC. Similar patterns were found in phase iv, the data increased by 4.81% and 4.96% respectively in ABS-SMC, while in BS-SMC increased by 8.40% and 17.53% respectively. So we can conclude that the time required to re-follow the desired trajectory by using ABS-SMC was reduced and the maximum error also remained small under uncertain resistances. The ABS-SMC can achieve a better anti-interference performance compared with the BS-SMC, attributing to its ability of estimating external disturbance and adjusting the control output accordingly.

To further verify superior ability of the proposed method, we also compared our results with other recently published works, which also aimed to control the PMs-driven rehabilitation robot. As summarized in Table 7, the proposed control method shows a better performance. Zhang et al. [53] used adaptive patient-cooperative control method to control a compliant ankle rehabilitation robot driven by PMs. They conducted the experiments with the subject, and the root mean square deviation (RMSD) was 2.34°. Jamwal et al. [18] used a fuzzy-based disturbance observer (FBDO) to control a 3-DOF ankle rehabilitation robot driven by PMs. The maximum error (ME) and average error (AE) of end-effector were 22.93% and 6.43%. The team also designed a robust iterative feedback tuning control scheme to improve the performance, and the ME and RMSD of trajectory tracking of the robot were about 12.48% and 1.40° [9]. In addition, Su at al. [54] proposed a model-based chattering mitigation robust variable control (CRVC) method and applied this method to control a lower limb rehabilitation robot driven by PMs. The ME of the end-effector was 15.00% and The RMSD was 2.34°. In this paper, when there was a participant, the ME, AE and RMSD were 7.05%, 2.15% and 0.78°, respectively. It can be seen from the above analysis that the control performance of the proposed method is obviously better than that of the above methods. 

From the experimental results analysis, it can be concluded that the ABS-SMC estimates external disturbance and adaptively adjusts the control law so the performance is obviously better than that of the BS-SMC and the recent published control schemes in [15,23,43,44] in terms of response speed, control accuracy, robustness and ability to resist external disturbance. This controller can meet the rehabilitation demands of patients under dynamic conditions.

## 5. Conclusions

In this paper, a 2-DOF parallel robot was developed for ankle rehabilitation and the inverse kinematics model as well as the dynamics model of the robot were constructed. This paper proposed an ABS-SMC for PMs by introducing a disturbance observer, so the external disturbances can be estimated and the control output can be adjusted in real time. Experimental results show that the ABS-SMC had better trajectory tracking performance compared with the conventional method. The proposed method can greatly reduce chattering, which may reduce secondary damage to the patient. When participants were involved, the tracking error of traditional method obviously increased while the error of the proposed method remained small. In addition, the ABS-SMC has a better anti-interference ability. When the ankle rehabilitation robot was applied with greater resistance, the proposed method could quickly track the desired trajectory after removing the resistance. How the control would perform under uncertainties in the model and the applied torque is also need to be studied in the future. Because of the complexity of the ankle rehabilitation robot, it is difficult to establish a precise dynamic model. Our model here can match the real system to a large extent, which can also be reflected from the experimental results. However, the model uncertainties should be optimized further and the applied torque can be measured in real time by using a force/torque sensor to reach a more accurate model that will in turn improve the control performance. To improve the patient’s participation in the future work, patient force feedback must be considered. In this case, the performance of current position/force hybrid control and impedance control can be improved by incorporating the proposed ABS-SMC method. Furthermore, functional electrical stimulation, and biological signals should also be applied to the control of the robot to improve the patient’s voluntary participation and rehabilitation training performance.

## Figures and Tables

**Table 1:** Statistical analysis of actuator position tracking errors under different control methods (without subject).

	Methods	Maximum Error (mm)			Average Error (mm)		
		A1	A2	A3	A1	A2	A3
Position tracking results	ABS-SMC	0.84	1.05	0.93	0.39	0.47	0.46
	BS-SMC	1.48	1.64	1.55	0.64	0.72	0.75

**Table 2:** Statistical analysis of end-effector angle tracking errors under different control methods (without subject).

	Methods	Maximum Error (°)		Average Error (°)	
		*θ*	*φ*	*θ*	*φ*
Angle tracking results	ABS-SMC	0.69	0.68	0.19	0.20
	BS-SMC	1.48	1.41	0.44	0.44

**Table 3:** Information of all involved subject.

Participants	Gender	Age	Height (cm)	Weight (kg)
Subject 1	male	23	175	65
Subject 2	male	22	178	64
Subject 3	female	23	160	49
Subject 4	female	24	165	50
Subject 5	male	25	180	70

**Table 4:** Statistical analysis of actuator position tracking errors under different control methods (with five subjects).

	Participants	Methods	Maximum Error (mm)			Average Error (mm)		
			A1	A2	A3	A1	A2	A3
Position tracking results	Subject 1	ABS-SMC	1.10	1.13	1.33	0.43	0.47	0.49
		BS-SMC	2.71	3.60	3.24	1.30	1.48	1.56
	Subject 2	ABS-SMC	1.52	2.07	1.76	0.39	0.47	0.37
		BS-SMC	3.71	4.67	4.20	1.19	1.43	1.07
	Subject 3	ABS-SMC	1.53	2.02	1.81	0.40	0.47	0.37
		BS-SMC	3.90	5.01	4.19	1.17	1.46	1.10
	Subject 4	ABS-SMC	1.77	2.07	1.88	0.39	0.48	0.38
		BS-SMC	3.86	5.22	5.30	1.22	1.27	1.29
	Subject 5	ABS-SMC	1.39	1.97	1.66	0.39	0.47	0.37
		BS-SMC	3.74	4.96	4.63	1.14	1.34	1.09

**Table 5:** End-effector angle tracking errors under different control methods (with five subjects).

	Participants	Methods	Maximum Error (°)		Average Error (°)	
			*θ*	*φ*	*θ*	*φ*
Angle tracking results	Subject 1	ABS-SMC	0.90	0.99	0.20	0.39
		BS-SMC	2.04	2.50	0.54	0.75
	Subject 2	ABS-SMC	1.12	0.99	0.29	0.28
		BS-SMC	2.25	2.18	0.50	0.78
	Subject 3	ABS-SMC	1.21	1.18	0.29	0.34
		BS-SMC	2.91	2.36	0.67	0.78
	Subject 4	ABS-SMC	1.41	1.13	0.43	0.34
		BS-SMC	3.32	2.75	0.63	0.66
	Subject 5	ABS-SMC	1.14	0.89	0.27	0.28
		BS-SMC	2.97	2.17	0.92	0.94

**Table 6:** Resistance force and duration of four phases in the experiment.

	Man-Made Resistance	Size (N)	Duration (s)
Phase i (P i)	None	0	0
Phase ii (P ii)	Applied	10	2
Phase iii (P iii)	Applied	30	2
Phase iv (P iv)	Applied	30	3

**Table 7:** Comparison of existing control methods and the proposed method for PMs-driven parallel rehabilitation robot. (*, unknown).

Literature	End-Effector Tracking Error					
	Without Human Participant			With Human Participant		
	ME (%)	AE (%)	RMSD	ME (%)	AE (%)	RMSD
[9]	11.18	*	1.35	12.48	*	1.40
[18]	*	*	*	22.93	6.43	*
[53]	*	*	*	*	*	2.34
[54]	*	*	*	15.00	*	*
Current study	3.45	1.00	0.44	7.05	2.15	0.78
